# Genome-wide identification of TCP transcription factors and their potential roles in hydrolyzable tannin production in *Quercus variabilis* cupule

**DOI:** 10.3389/fpls.2024.1444081

**Published:** 2024-08-06

**Authors:** Yaochen Wang, Jinjin Li, Yixin Chen, Zhaowei Yu, Puyuan Liu, Guolei Li, Qinsong Yang

**Affiliations:** ^1^ Research Center of Deciduous Oaks, Beijing Forestry University, Beijing, China; ^2^ Deciduous Oak Improvement and Regeneration Innovation Team of State Forestry and Grassland Administration, Beijing Forestry University, Beijing, China; ^3^ Key Laboratory for Silviculture and Conservation, Ministry of Education, Beijing Forestry University, Beijing, China

**Keywords:** *Quercus variabilis*, traditional medicine, hydrolyzable tannins, TCP gene family, UGT84A13

## Abstract

Hydrolyzable tannins (HTs) have garnered significant attention due to their proven beneficial effects in the clinical treatment of various diseases. The cupule of Chinese cork oak (*Quercus variabilis* Blume) has been used as raw material of traditional medicine for centuries for its high content of HTs. Previous studies have identified UGT84A13 as a key enzyme in the HT biosynthesis pathway in *Q. variabilis*, but the transcriptional regulation network of *UGT84A13* remains obscure. Here, we performed a comprehensive genome-wide identification of the TCP transcription factors in *Q. variabilis*, elucidating their molecular evolution and gene structure. Gene expression analysis showed that *TCP3* from the CIN subfamily and *TCP6* from the PCF subfamily were co-expressed with *UGT84A13* in cupule. Further functional characterization using dual-luciferase assays confirmed that TCP3, rather than TCP6, played a role in the transcriptional regulation of *UGT84A13*, thus promoting HT biosynthesis in the cupule of *Q. variabilis*. Our work identified TCP family members in *Q. variabilis* for the first time, and provided novel insights into the transcriptional regulatory network of *UGT84A13* and HT biosynthesis in *Q. variabilis*, explaining the reason why the cupule enriches HTs that could be used for traditional medicine.

## 1 Introduction

Tannins are water-soluble polyphenolic compounds synthesized by plants through secondary metabolism. They are produced via the biological inheritance pathway from galloyl esters or low polymerized proanthocyanidins (Lewis and Yamamoto, 1989). As plant defense chemicals, tannins protect plants from fungi, pathogens, and herbivores (Gallardo et al., 2019; Xie et al., 2019; Das et al., 2020). Based on their phenolic group connections, tannins are categorized as hydrolyzable tannins (HTs) and condensed tannins (CTs). For human health, HTs exhibit antioxidant and antimicrobial effects, showing potential as effective natural antibiotics (Zhou et al., 2019). Additionally, HTs can relax vascular smooth muscle and are effective against arterial hypertension (Marrone et al., 2023). HTs can treat diabetic complications by acting on the hexosamine pathway (Laddha and Kulkarni, 2019). HTs can alleviate stress-induced depression by modulating cortisol and monoamine neurotransmitter levels, reducing oxidative stress, and potentially serving as a complementary treatment for depression (Chandrasekhar et al., 2017). Interest in utilizing natural HTs as potential nutritional supplements or adjunct therapies is increasing (Melo et al., 2023). Plants such as pomegranate, chestnut, and oak species are rich sources of HTs (Deng, 2018; Habashi et al., 2019; Sun et al., 2021; Yang et al., 2023).

Due to the widespread application of HTs, there is significant interest in their biosynthetic pathway. However, model plants including Arabidopsis, rice, and poplar do not contain abundant levels of HTs, leading to uncertainties in the regulatory pathways of HT biosynthesis. Previous studies have indicated that HT synthesis relies on the catalysis of dehydroquinate dehydratase/shikimate dehydrogenase (DQD/SDH) and UDP-dependent glycosyltransferase (UGT). DQD/SDHs have been identified as enzymes involved in the biosynthesis of gallic acid, the precursor to HT production. In *Vitis vinifera*, *VvSDH3* and *VvSDH4* are involved in gallic acid biosynthesis (Bontpart et al., 2016). In *Eucalyptus camaldulensis*, EcDQD/SDH2 and EcDQD/SDH3 are involved in gallate formation, dehydroquinate dehydratase activity, and shikimate dehydrogenase activity, resulting in the accumulation of HTs which significantly enhances aluminum tolerance (Tahara et al., 2021). In *Q. variabilis, UGT*, rather than *SDH*, exhibits tissue-specific expression (Yang et al., 2024). β-glucogallin, as a biomarker of HTs in oak cupule, is formed by the combination of UDP-glucose and gallic acid, with UGT being the critical enzyme in this process (Niemetz and Gross, 2005; Tahara et al., 2021; Yang et al., 2024). Expression patterns and correlation analyses in *Canarium album* L. have revealed that the highly expressed *CaUGT84A77* can catalyze the production of β-glucogallin from gallic acid (Ye et al., 2021). UGT84A13 is a pivotal enzyme in the biosynthesis of β-glucogallin in oak species, further influencing HT synthesis, as confirmed in *Q. robur* and *Q. variabilis* (Mittasch et al., 2014; Yang et al., 2024). However, the transcriptional regulation of these UGTs remains largely unknown. Our previous research found that *Q. variabilis* WRKY32/59 and HB26 can activate the expression of *UGT84A13* (Yang et al., 2023, 2024). However, other transcription factors such as ERFs, TCP, and bHLH may also be regulatory factors in HT biosynthesis, and the molecular regulatory network involving UGT has yet to be established.

TCP family is a class of transcription factors widely present in plants, named after their prototypical members Teosinte branched (TB1), Cycloidea (CYC), and Proliferating Cell Factor (PCF) (Cubas et al., 1999). The TCP family contains an atypical basic-helix-loop-helix (bHLH) secondary structure composed of 55-60 amino acid residues, known as the TCP conserved domain. This domain can bind to DNA and interact with other proteins to regulate the transcription of target genes (Aggarwal et al., 2010). Based on differences in the conserved domain, the TCP family is divided into Class I (PCF subfamily) and Class II (CIN and CYC/TB1 subfamilies) (Lin et al., 2016). TCPs are broadly involved in various physiological processes of plant growth and development, including seed germination (Tatematsu et al., 2008), bud dormancy (Wang et al., 2020), fruit development (Xie et al., 2020), response to abiotic stress (Xu et al., 2021), and secondary metabolism. Overexpression of *AtTCP4* can promote the expression of the *LOX2* gene, leading to the accumulation of jasmonic acid and accelerating leaf senescence (Li et al., 2024). PeTCP10 from *Phyllostachys edulis*, when transformed into Arabidopsis, significantly enhances the salt tolerance of transgenic Arabidopsis during the vegetative growth phase by promoting catalase activity to improve antioxidant capacity (Xu et al., 2021). miRNA319-mediated silencing of the transcription factor OsTCP21 and its target genes blocks JA signal transduction to manipulate rice immune responses (Zhang et al., 2018). However, it remains unclear whether TCP transcription factors are involved in the biosynthesis of HTs.

Chinese cork oak (*Quercus variabilis*) is a dominant and resource-worthy species in East Asian (Cao and Chen, 2015; Hu et al., 2016). The cupule of *Q. variabilis* has been used as raw material for traditional medicine for centuries. Traditionally, its branches and cupules are used in Chinese medicine to treat malignant sores and diarrhea (Jia et al., 2013). The ethanolic extract of *Q. variabilis* cupules exhibits significant inhibitory effects on *Salmonella paratyphi A* and *Staphylococcus aureus*, surpassing the activity of pomegranate peel ethanolic extract and ellagic acid (Zhou, 2016). Modern research indicates that the main active components of *Q. variabilis* extracts are HTs (Yang et al., 2023). However, the transcriptional regulation of HT biosynthesis still remains largely unknown.

In this study, we comprehensively analyzed *TCP* family genes in *Q. variabilis* genome. Combined with transcriptome analysis of different tissues and organs of *Q. variabilis*, we explored the regulatory function of the *TCP* family in HTs biosynthesis. Dual-luciferase assays were conducted to investigate the regulatory functions of the *TCP* gene family in HTs production in *Q. variabilis*. To our knowledge, this research is the first comprehensive analysis of *TCP* genes in *Q. variabilis*. It has also refined the molecular regulatory network regulating HT biosynthesis in *Q. variabilis* involving *UGT84A13*, explaining the reason why the cupule enriches HTs that could be used for traditional medicine.

## 2 Materials and methods

### 2.1 Identification of *TCP* gene family members

The Hidden Markov Model (HMM) file was downloaded from the Pfam database (https://pfam.xfam.org/) using the protein accession number PF03634. We then used the protein sequences of the Arabidopsis *TCP* gene family for sequence alignment to identify and preliminarily screen the *TCP* gene family. Employing HMMER3 software with the HMM domain of TCP as the query, we searched the entire protein sequence database of the *Q. variabilis* genome (Yang et al., 2024). Additionally, we downloaded amino acid sequences of Arabidopsis *TCP* gene family members from TAIR11 (https://www.arabidopsis.org/) and used them as query sequences in BLAST comparisons within TBtools. We utilized the *Q. variabilis* whole-genome protein sequences as the reference library, setting an E-value threshold of 1e^-5^. The sequences obtained from both methods were integrated, leading to the identification of 22 *TCP* family genes. We used the Gene Location plugin in TBtools along with the annotation data of the *Q. variabilis* genome and the ID numbers of the target genes to determine the relative chromosomal positions and gene density on each chromosome. We then visualized the chromosomal distribution. The physicochemical properties of the *TCP* gene family were assessed using the Protein Parameter Calc plugin in TBtools. The amino acid sequences of the identified *TCP* gene family were then modeled using the SWISS-MODEL online platform (https://swissmodel.expasy.org/). Upon reviewing multiple results, the optimal protein structure model was selected based on the GMQE score (ranging from 0 to 1, with higher values indicating greater reliability) and the QMEAN score (ranging from -4 to 0, with values closer to 0 indicating a higher degree of similarity between the target and template proteins).

### 2.2 Evolutionary analysis of the *TCP* gene family

We constructed an evolutionary tree using the full-length protein sequences from 24 *Arabidopsis thaliana TCP* gene family members, 13 *Quercus robur TCP* gene family members, and 22 *TCP* gene family members. The analysis was performed using MEGA 7.0 software (Kumar et al., 2016), with settings including the Neighbor-Joining (NJ) method, a bootstrap value of 1000, the Poisson model, and pairwise deletion. Subfamily classification of the *TCP* gene family members was based on established methods used for the Arabidopsis *TCP* gene family (Yao et al., 2007). The evolutionary tree was visually refined using the Chiplot online tool (https://www.chiplot.online/#) (Xie et al., 2023).

### 2.3 Motif analysis and gene structure of the *TCP* gene family

Motif analysis was conducted using the MEME online tool (http://memesuite.org/tools/meme), specifying the identification of 6 motifs. The results were then imported into TBtools software for visualization of conserved protein motifs. The annotation file for the *Q. variabilis* genome, along with the IDs of its *TCP* gene family members, was uploaded into TBtools (Chen et al., 2023) to generate a visual representation of their gene structures.

### 2.4 Promoter cis-element analysis of the *TCP* gene family

The TBtools software was used to extract the 2000 bp upstream sequence of the *TCP* genes’ CDS regions. Cis-elements within the *TCP* promoters were identified using the PlantCARE database (https://bioinformatics.psb.ugent).Visualization of the data was performed using TBtools software (Chen et al., 2023).

### 2.5 Intraspecific collinearity analysis of the *TCP* gene family

We used the MCScanX (Wang et al., 2012) plugin in TBtools (Chen et al., 2023) to calculate and analyze collinearity within the *Q. variabilis* genome, and between *A. thaliana* and *Q. variabilis*, using default parameter settings. The collinearity analysis results were visualized using the Dual Synteny Plot plugin within TBtools (Chen et al., 2023).

### 2.6 Expression pattern analysis of *TCP* genes in different organs

To identify *TCP* genes potentially involved in the transcriptional regulatory network of *UGT84A13* and its regulation of HTs, we analyzed our published transcriptome data from various tissues of *Q. variabilis* (Yang et al., 2023). This analysis provided expression profiles of *TCP* genes across different tissues. We integrated the expression levels of *UGT84A13* and the content data of HTs in various tissues generated in our pervious study (Yang et al., 2023). We also analyzed the candidate *TCP* genes, along with the previously reported *HB26*, *WRKY32*, and *WRKY59* associated with HT biosynthesis (Yang et al., 2023, 2024), to investigate potential correlations in their expression. Normalization and row standardization were performed using the HeatMap plugin in TBtools software, and the results were visualized (Chen et al., 2023).

### 2.7 Bioinformatics platform predicts TCP transcription factors

We utilized JASPAR 2024 (https://jaspar.elixir.no/analysis) (Rauluseviciute et al., 2024) to make predictions with the TCP template in the database. The promoter region of *UGT84A13* was inputted into the database for predicting TCP transcription factor binding sites, with the relative profile score threshold set at 80%. Potential binding sites were selected based on their scores.

### 2.8 Dual luciferase assay

To further elucidate the mechanism of direct interaction between TCP and *UGT84A13*, a dual-luciferase reporter assay was conducted as previously described (Yang et al., 2020). The complete CDSs of TCP3 and TCP6 were cloned into the pGreen II 0029 62-SK vector, while the *UGT84A13* promoter, encompassing 2000 bp upstream, was cloned into the pGreen II 0800-LUC vector. These constructs were introduced into Agrobacterium tumefaciens strain GV3101 cells containing the pSoup vector. Following the method outlined by Yang et al. (2020), the optical density (OD) of the SK and LUC bacterial suspensions was adjusted to 1.0, mixed in a 10:1 ratio, and the mixture was used to infect tobacco leaves. After 56 hours of infection, the dual-LUC assays were performed using the Dual Luciferase Reporter Assay Kit (Vazyme, Nanjing, China) as instructed. Fluorescent signals were measured using a Glomax Discover and Explorer (Promega, USA). The experiment was conducted with six biological replicates.

## 3 Results

### 3.1 Genome-wide identification of *TCP* genes

**Figure 1:**
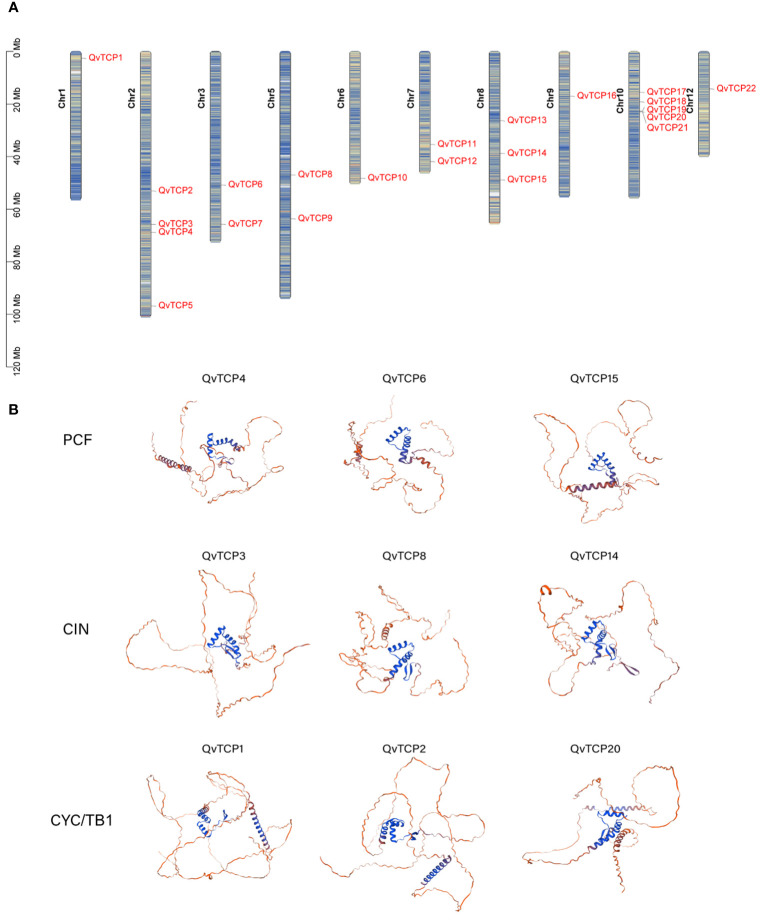
**(A)** Shows the distribution of the *TCP* gene on the chromosome in *Q. variabilis*. The color blocks, ranging from blue to red, indicate a gradual increase in gene density. **(B)** Predicted protein structure models of *TCP* gene family in *Q. variabilis*. In this figure, three members of each subfamily were randomly selected for display, and the complete TCP family structure prediction is shown in **Supplementary Figure S1**. The predicted protein structure models of the TCP family in *Q. variabilis* are displayed. The blue color signifies regions where the prediction results are considered highly reliable, while the red color signifies regions with lower reliability in the prediction results.

By using BLAST and HMMER search, we identified a total of 22 *TCP* genes in the genome of *Q. variabilis* for further analysis. Chromosomal localization analysis (**Figure 1A**) revealed that these genes are distributed across chromosomes 1, 2, 3, 5, 6, 7, 8, 9, 10, and 12. The 22 *TCP* genes were sequentially renamed from *TCP1* to *TCP22* based on their chromosomal positions.

Physicochemical property analysis (**Supplementary Table S1**) indicated that the amino acid sequences encoded by the *TCP* genes range from 211 amino acids (TCP12) to 566 amino acids (TCP5). The isoelectric points span from 6.05 (TCP21) to 9.72 (TCP10), and the molecular weights range from 22.44 kDa (TCP12) to 60.16 kDa (TCP5). The instability index of TCP proteins varies between 34.72 (TCP20) and 67.46 (TCP17). Additionally, the aliphatic index ranges from 54.08 (TCP5) to 76.52 (TCP2), and the average hydrophilicity coefficient ranges from -0.930 (TCP21) to -0.226 (TCP11), suggesting that TCP proteins are all hydrophilic.

Prediction of the tertiary structures of the TCP proteins (**Figure 1B**) showed that their structures mainly consist of random coils and an atypical bHLH domain. The bHLH domain forms a reverse parallel conformation with two helices, and the predicted TCP protein structure indicates that the angle between the helices is nearly perpendicular, similar to the helical topology of the RHH domain. These structural characteristics are consistent with those observed in TCP proteins from other species such as rice and Arabidopsis (Sun et al., 2020).

### 3.2 Evolutionary analysis of *TCP* genes

**Figure 2:**
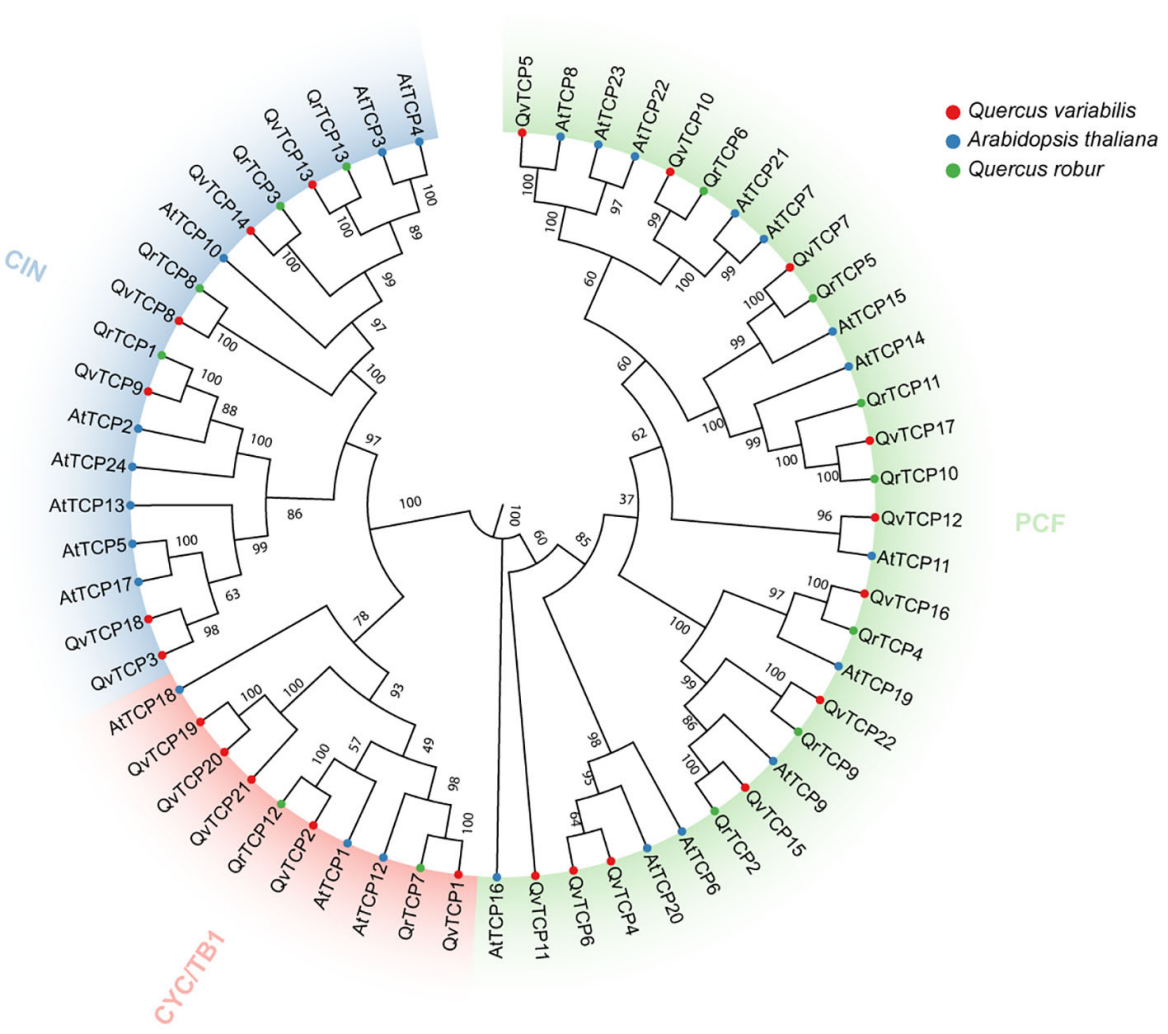
Evolutionary analysis of 59 *TCP* family genes from *Q. variabilis*, *A. thaliana* and *Q. robur* based on their protein alignment. The evolutionary tree was constructed using the NJ method, with 1000 bootstrap replicates in MEGA. The *TCP* genes were clustered into three clades (CYC/TB1, CIN and PCF), each one colored differently. The less distance between genes, the higher the similarity in the motifs they contain, suggesting potential shared biological functions.

To further elucidate the evolutionary relationships among *TCP* genes in *Q. variabilis* and other species, we constructed an evolutionary tree using 22 identified TCP protein sequences from *Q. variabilis*, 24 from *A. thaliana*, and 13 from *Quercus robur* (**Figure 2**). Based on the classification of the Arabidopsis *TCP* gene family, *TCP* family members were categorized into three subfamilies: CYC/TB1, CIN, and PCF (Yao et al., 2007). The CYC/TB1 subfamily contains 5 members, CIN includes 6 members, and PCF comprises the largest number with 11 members. In *Q. robur*, the CYC/TB1 subfamily has 2 members, CIN has 4 members, and PCF, the largest subfamily, has 7 members. In *A. thaliana*, CYC/TB1 contains 8 members, CIN has 3 members, and PCF, with the most members, has 13. In the evolutionary tree, shorter distances between genes indicate higher similarity in their motifs, suggesting they may share similar biological functions.

### 3.3 Motif analysis and gene structure of TCPs

**Figure 3:**
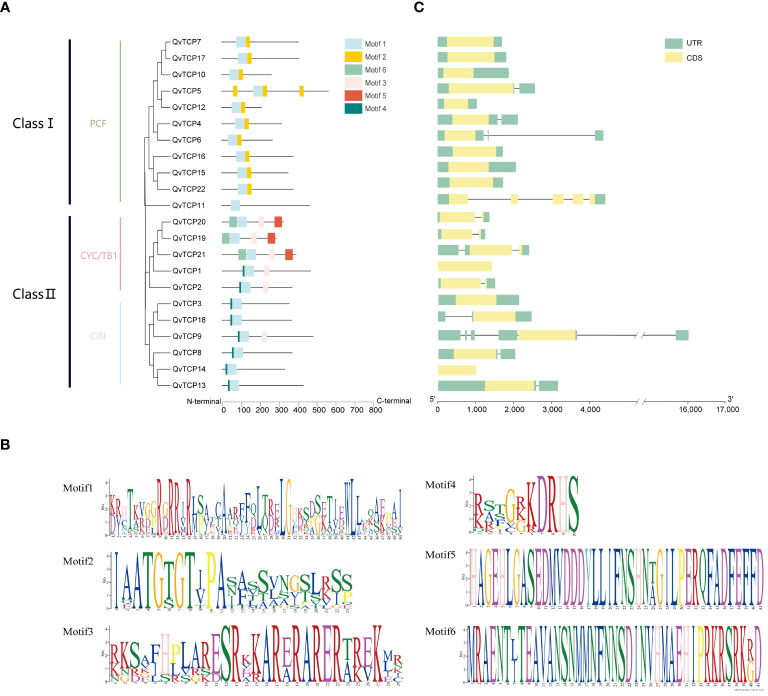
**(A)** Protein motif and gene structure analysis of *TCP* family genes identified in *Q. variabilis*. The evolutionary tree of TCP protein sequences is shown, with conserved protein motifs colored differently. **(B)** Exon-intron distribution analysis of *TCP* genes. The green boxes represent coding sequences (CDS), the green boxes represent untranslated regions (UTR), and the black lines represent intron positions, respectively. **(C)** The conserved motifs of the TCP family members in *Q. variabilis*.

To analyze the conservation of the TCP family protein sequences, we visualized the gene structure using TBtools software. Based on MEME software, six conserved motifs were identified within the protein sequences. Motif 1 is present in all TCPs and is similarly positioned (**Figures 3A, B**). Upon alignment, it was found that the sequence of Motif 1 has a high degree of consistency with the TCP conserved domain sequence. Protein sequences within the same subfamily are more similar. In the Class I subfamily, Motif 1 and Motif 2 appear exclusively in combination, while in the Class II subfamily, Motif 1 and Motif 4 are exclusively combined. Motif 5 and Motif 6 are only present in the CYC/TB1 subgroup of Class II and are found only in proteins TCP19, TCP20, and TCP21.From this, it can be deduced that each subfamily has its unique combination of motifs, and the conserved motifs within the same subfamily are very similar or identical. This analysis is consistent with our evolutionary tree analysis, indicating significant differences in conserved motifs among TCP proteins from different subfamilies, suggesting distinct functional roles among members of different subfamilies.

To explore the diversity of *TCP* gene structures, we conducted a gene structure analysis. The results (**Figure 3C**) show significant variation in the number of exons and introns among the 22 *TCP* genes. Generally, genes that cluster together have similar gene structures, and homologous genes within the same branch exhibit consistent structures, suggesting that members of the same subfamily may have similar biological functions. All *TCP* gene regions contain introns, with the number of exons ranging from 1 to 6 and introns from 1 to 5. Most *TCP* genes have relatively simple structures, though a few genes, such as *TCP9*, have intron lengths significantly different from others. The consistent structures of homologous genes within the same branch further suggest that members of the same subfamily may have similar biological functions.

### 3.4 Cis-acting elements analysis of *TCP* genes

**Figure 4:**
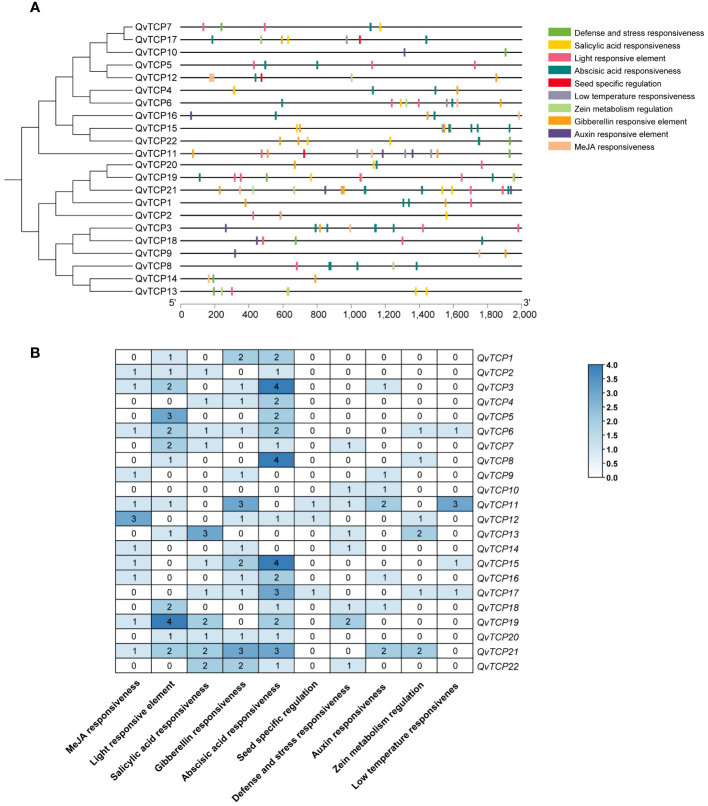
Cis-acting element distribution of *TCP* gene family in *Q. variabilis*. **(A)** Analysis of cis-regulatory elements within *TCP* gene promoters. **(B)** The numerical values and colors represent the number of cis-elements identified within each *TCP* gene promoter.

Promoters play a regulatory role in the transcription and expression of genes. To better understand the biological functions and mechanisms of *TCP* genes, this study analyzed the genomic sequences of 2,000 bp upstream of the coding regions of the *TCP* genes, as potential promoter regions. Ten important cis-acting elements were identified (**Figure 4**). These elements are primarily classified into hormone responses, environmental responses, abiotic stress responses, and stress responses. Hormone response elements, such as those for gibberellins, abscisic acid, methyl jasmonate, salicylic acid, auxin, and zeatin, are widely present in the promoters of the *TCP* gene family, indicating that *TCP* family genes are closely related to hormone signal regulation. Among the stress-related elements, some *TCPs* contain cis-acting elements responsive to cold stress, defense, and stress, suggesting that these *TCPs* play roles in resistance to and response to cold stress and adversity. Additionally, light-responsive elements are widely distributed across the TCP family, suggesting that the *TCP* gene family may be regulated by light signals. All 22 *TCP* genes contain one or more types of cis-elements involved in various functions, implying that *TCP* family genes may participate in multiple regulatory aspects during plant growth and development. Further research revealed that members of both Class I and Class II subfamilies are generally involved in the aforementioned cis-elements, although there are some differences in the number of gene members associated with each type of cis-element.

### 3.5 Evolutionary analysis of the *TCP* family

**Figure 5:**
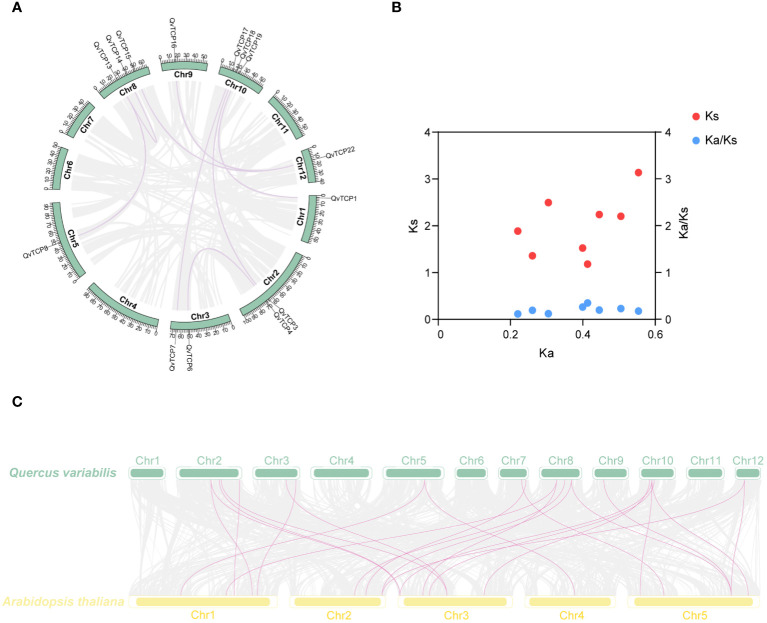
**(A)** Shows a schematic diagram of the motif and gene structure of *TCP* family members. The black vertical lines on the chromosomes indicate gene localization, while the inner lines represent collinearity. Gray lines indicate genome-wide collinearity, and purple lines represent the colinear region of *TCP*s. **(B)** Ka, Ks, Ka/Ks ratio calculation of the paralog pairs of *TCP*s in *Q. variabilis.* **(C)** Collinear analysis of *TCP* gene family between *A. thaliana* and *Q. variabilis*.

To detect instances of gene duplication within the *TCP* family, we conducted a syntenic analysis across the *Q. variabilis* genome, focusing on the *TCP* family. This is illustrated by the purple lines representing syntenic relationships (**Figure 5A**). These collinear gene pairs may have originated from the single ancient whole-genome duplication (Yang et al., 2024). We identified a total of eight syntenic gene pairs (**Supplementary Table S3**) and eight syntenic blocks and, the Ka/Ks values for all eight syntenic gene pairs ranged from 0.1 to 0.4 (**Supplementary Table S4**; **Figure 5B**), indicating that the expansion of the *TCP* family has likely been subject to purifying selection, which eliminates deleterious non-synonymous mutations during the natural selection process in species evolution.

To examine the evolution of the *Q. variabilis TCP* gene family, we performed a syntenic analysis between the *TCP* gene families of *Q. variabilis* and *A. thaliana*, identifying 24 syntenic gene pairs (**Figure 5C**). With 22 members within the *TCP* gene family, this result suggested that the majority of the family members were highly conserved throughout the evolutionary process.

### 3.6 Expression profiles of *TCP* genes and identification of candidates for HT biosynthesis

**Figure 6:**
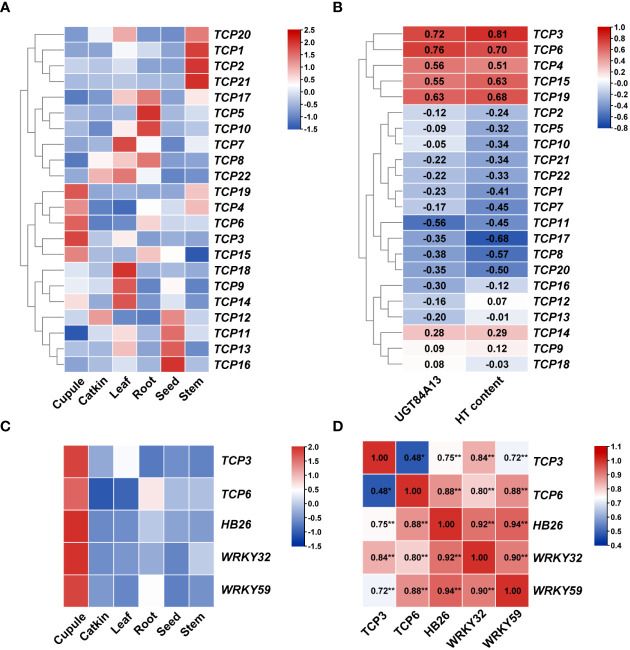
**(A)** The expression patterns of *TCP* family members in various organs of *Q. variabilis*. Red blocks indicate a relatively high level of TPM values, while blue blocks indicate a relatively low level of TPM values after normalization. The red gene name indicates the *TCP* genes that is highly expressed specifically in the cupule. **(B)** Correlation of *TCP* genes with *UGT84A13* expression and HT content. **(C)** The expression patterns of genes associated with HT biosynthesis in various organs of *Q. variabilis*. **(D)** Correlation of genes associated with HT biosynthesis expression. *Significant correlation at the 0.05 level; **Significant correlation at the 0.01 level.

Gene expression patterns are often closely linked to their functions, making the analysis of these patterns crucial for understanding gene function. To explore the expression of *TCP* genes across various tissues and to further comprehend the impact of the *TCP* gene family on HT content and related mechanisms in *Q. variabilis* tissues, we analyzed the expression patterns of *TCP* family genes using heatmaps and transcriptional data (Transcripts Per Million, TPM). The transcriptional data were log-transformed and row-normalized to generate heatmaps and perform clustering (**Figure 6A**). Given that the expression variation of *UGT84A13* is a primary factor contributing to the differential accumulation of β-glucogallin and HTs (Yang et al., 2024), we screened for TCP transcription factors that are specifically highly expressed in cupule and have high TPM values in the cupule. Preliminary screening identified five transcription factors: *TCP3*, *TCP4*, *TCP6*, *TCP15*, and *TCP19*. Additionally, we conducted a correlation analysis between the expression levels of *TCP* and *UGT84A13*, as well as the abundance of HTs measured in our previous study (Yang et al., 2023). The results indicated that the expression levels of two candidate genes (**Figure 6B**), *TCP3* and *TCP6*, were significantly correlated (*p* < 0.001) with the expression level of *UGT84A13*. Expression patterns of genes associated with HT biosynthesis in different organs of *Q. variabilis* (**Figure 6C**) revealed that these genes were highly expressed in the cupule. We conducted correlation analyses between the expression levels of *TCP3/6* and genes associated with HT biosynthesis (**Figure 6D**). The results indicated a significant correlation between the expression levels of *TCP3* and *TCP6* and those of *HB26* and *WRKY32/59* (p < 0.001). Therefore, we identified *TCP3* and *TCP6* as potential candidate transcription factors that may regulate the biosynthesis of HTs in the cupule.

### 3.7 Dual luciferase assay

**Figure 7:**
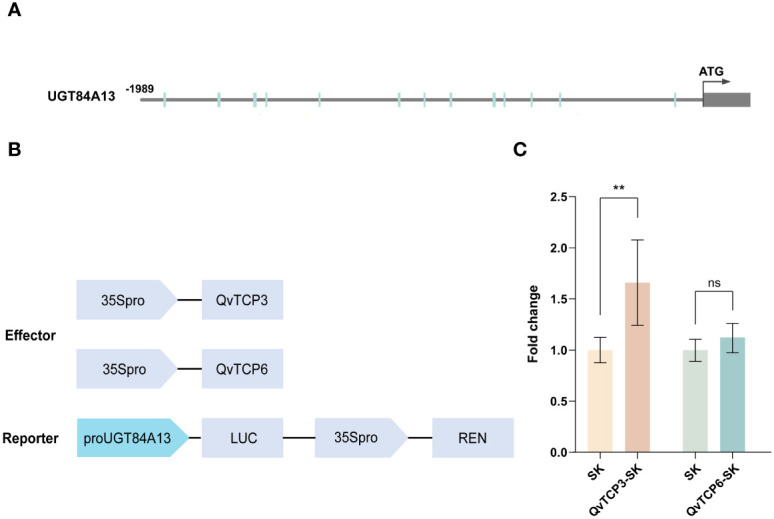
**(A)** JASPAR predicts the potential binding sites of TCP transcription factors in the *UGT84A13* promoter. **(B)** Sketch map of the vectors used in dual-luciferase assays. **(C)** The dual luciferase assay for *QvTCP3* and *QvTCP6*. Error bars represent the standard deviation of 6 biological replicates. Asterisks indicate a significant difference between the experimental group and the control group, while ns denotes no significant difference (Student’s *t*-test; **, *p* < 0.01).

JASPAR was used to predict a number of potential binding sites on the *UGT84A13* promoter where the TCP transcription factors could bind (**Figure 7A**). To verify the role of TCP3 and TCP6 in regulating *UGT84A13*, we cloned the full-length CDS (coding sequence) of these two genes into SK vector (**Figure 7B**). The co-expression of proUGT84A13-LUC and QvTCP3-SK significantly increased luciferase activity (**Figure 7C**); However, there was no significant change with QvTCP6-SK (**Figure 7C**). These results confirmed that TCP3, rather than TCP6, interacted with the promoter of *UGT84A13* and activated its expression, suggesting the potential role of TCP transcription factors in HT biosynthesis.

## 4 Discussion

Tannins, also known as plant polyphenols, are widely found in the leaves, bark, roots, and fruits of plants (Shirmohammadli et al., 2018). They constitute an important class of secondary metabolites in plants, providing protection against herbivory and enhancing resistance to pests and diseases (Tondi et al., 2013). Traditionally, tannins are classified into two main types: condensed tannins and hydrolyzable tannins (Freudenberg, 1960). HTs possess anti-inflammatory and immunomodulatory functions, can lower blood sugar and lipids, have antioxidant capabilities, and exhibit DNA repair and anti-cancer effects (Smeriglio et al., 2017). Additionally, the ester bonds of HTs can be hydrolyzed by human digestive enzymes, making them easier to break down into smaller molecules after ingestion. These molecules are then metabolized by intestinal microbiota and absorbed in the intestines (Smeriglio et al., 2017), making HTs an ideal health supplement and nutritional aid. HTs are mainly found in oak, grape seeds, pomegranate peels, and other sources that are not easily supplemented through a daily diet (Fischer et al., 2011). Therefore, a plant rich in HTs is needed, and methods to extract HTs from it must be devised to meet demand. Studies have found that the tannins in oak species are mainly HTs (Yin et al., 2019). Oaks have garnered attention due to their high tannin content in tissues, large reserves, and easy access to raw materials. Among these, *Q. variabilis* has a higher HT content than other oak species such as *Q. aliena* and *Q. dentata* (Yang et al., 2024), indicating that *Q. variabilis* is an ideal material for extracting HTs.

Compared to the well-studied CTs (Saigo et al., 2020), the biosynthetic regulatory pathways of HTs remain incompletely understood. Current research indicates that the expression of the key enzyme, UDP-glucose transferase (UGT), determines the synthesis of HTs (Mittasch et al., 2014). Additionally, UGT is an essential enzyme for the biosynthesis of HTs in plants such as pomegranates (Yuan et al., 2018). UGT84A13s are pivotal enzymes in the HT synthesis pathway of oak genus plants (Mittasch et al., 2014), capable of catalyzing gallic acid into β-glucogallin. β-glucogallin, a precursor of HT synthesis, possesses numerous pharmacological activities, including antioxidant and anti-inflammatory effects, anti-diabetic properties, prevention of diabetic retinopathy complications, protection against eye diseases, and defense against ultraviolet radiation (Khan et al., 2022; Rohilla et al., 2023). However, the regulation of *UGT84A13* in the HT biosynthetic pathway of oaks has not been fully elucidated, impeding further improvement in HT production efficiency. Our preliminary research identified 14 transcription factors that co-express significantly with *UGT84A13*, among which the role of *TCP* genes in the HT synthesis pathway remains unknown (Yang et al., 2024). In a study on the de-astringency of persimmon fruits, it was found that the expression of 2 TCP transcription factors was significantly suppressed during the reduction of HT content following maturation (Deng, 2018). In this study, we identified the entire genome of the *TCP* gene family and selected 2 members, *TCP3* and *TCP6*, which may regulate *UGT84A13*. The identification of the *TCP* gene family will provide more information for understanding the characteristics and potential capabilities of this plant-specific transcription factor.

TCP transcription factors are an ancient, conserved family of proteins widely distributed across various plant species, such as Arabidopsis, rice, and tomato (Cubas et al., 1999; Takeda et al., 2003; Parapunova et al., 2014). TCPs regulate plant morphogenesis and environmental adaptation by participating in multiple growth and developmental processes. In *Q. variabilis*, we identified a total of 22 *TCP* gene family members, which is fewer than the number of *TCP* genes identified in other higher plants, such as 24 in Arabidopsis, 52 in apple, 35 in peony, and 38 in cotton (Yao et al., 2007; Ma et al., 2014; Li et al., 2022; Tabarelli et al., 2022). This may suggest a contraction of the *TCP* gene family in *Q. variabilis*. Our study examined the characteristics of the TCP protein family, including the number of exons and introns, isoelectric points, and molecular weights. The TCP domain, consisting of about 60 amino acids and characterized by an atypical bHLH secondary structure, is a hallmark of TCP proteins that we analyzed. In addition to the conserved TCP domain, members of the TCP transcription factor family also possess other domains, such as the ECE domain (Howarth and Donoghue, 2006), which is found exclusively in the Class II CYC subgroup and whose function remains unclear and requires further investigation. Further research indicates that most genes within the same evolutionary branch have similar exon-intron compositions, suggesting a correlation between the genetic makeup of the *TCP* domain and its evolutionary history.The distribution of *TCP* genes is irregular, with the 22 *TCP* genes unevenly distributed across 10 *Q. variabilis* chromosomes (**Figure 1A**), and no copies of *TCP* genes were found on chromosomes 4 and 11. Based on the evolutionary analysis of Arabidopsis *TCP* genes, we divided the *TCP* family members into three subfamilies: CYC/TB1, CIN, and PCF, and constructed an evolutionary tree of TCP protein sequences in *Q. variabilis* (**Figure 2**).This allows us to further understand the potential functions of the *TCP* gene family. Evolutionarily, we found that the structure of *TCP* genes is closely related to their phylogeny. Members within the same subfamily have identical motif compositions, indicating that they have similar functions at the protein level.

According to reports, proteins from the CIN and PCF subfamilies of the *TCP* gene family are involved in responses to abiotic stresses (Xu et al., 2019; Hao et al., 2023). This aligns with the stress-resistant function of hydrolyzable tannins. It was found that TCP3 belongs to the CIN subfamily, while TCP6 belongs to the PCF subfamily (**Figure 2**). Studies have shown that defense-related plant hormones, including jasmonic acid (JA) and abscisic acid (ABA), regulate hydrolyzable tannins (HTs) in response to biotic and abiotic stresses (Iqbal and Poór, 2024). The application of exogenous ABA can increase the content of ellagic acid in the pericarp of *Vitis rotundifolia* (Sandhu et al., 2011). In studies on pomegranate, treatment with MeJA led to the accumulation of β-glucogallin in the leaves (Chang et al., 2021), and MeJA treatment increased the expression of *PgUGT84A23* and *PgUGT84A24*, affecting the content of HTs and confirming the key role of MeJA in HT production (Chang et al., 2021). Cis-element analysis of the TCP family indicates that the promoters of *TCP* genes contain various plant-specific binding elements, including those related to abiotic stress, such as ABA, MeJA, and SA (**Figure 4**). Numerous studies have shown that the TCP family responds to these plant hormones. In Arabidopsis, research confirmed that *TCP2* (CIN subfamily) positively regulates LOX2 promoter activity, directly regulating LOX2, an enzyme catalyzing a key step in JA biosynthesis (Schommer et al., 2008). In *Ginkgo biloba*, exogenous MeJA treatment significantly upregulated the expression of *GbTCP6* (CIN subfamily), *GbTCP11* (CIN subfamily), and *GbTCP13* (PCF subfamily) (Yu et al., 2022). In *Vitis vinifera* L., many members are strongly regulated by ABA treatment. Among them, *VvTCP14* (PCF subfamily) showed higher transcriptional expression levels after exogenous ABA treatment (Jiu et al., 2019). However, whether ABA or MeJA treatment regulates members of the *TCP* family and further increases the content of HTs in the cupule of *Q. variabilis* remains to be studied (**Figure 6A**). The correlation between TCP6 and other TFs identified in our previous studies was higher than that between TCP*3* and the other TFs, suggesting that TCP6 might indirectly activate *UGT84A13* through other TFs, which need further validation. However, the expression of *TCP3* in the cupules was much higher than that of *TCP6*. Considering the direct activation of TCP3 on *UGT84A13* promoter, we concluded that TCP3 is a key TF regulating the expression of *UGT84A13* and HT biosynthesis (**Figure 7**).

## 5 Conclusions

In summary, this study presents the first comprehensive genome-wide analysis of the *TCP* gene family in *Q. variabilis*. A total of 22 *TCP* genes were identified. Tissue-specific expression analysis and whole-genome identification of *TCP* genes led to the selection of *TCP3* and *TCP6* as candidate genes for regulating *UGT84A13* expression and HTs biosynthesis. A dual-luciferase assay confirmed that TCP3, but not TCP6, is involved in the transcriptional regulation of *UGT84A13*, consequently affecting HTs synthesis in *Q. variabilis*. This study provides new insights into the transcriptional regulation of *UGT84A13* and HTs biosynthesis, enhancing the understanding for the reason why the cupule of *Q. variabilis* could be used in medicine.

## Data availability statement

The RNA-Seq raw data can be obtained from CNGB Sequence Archive (CNSA) of China National GeneBank DataBase (CNGBdb, https://db.cngb.org/) with accession nos.:CNP0003737. Genome of *Q. variabilis* can be obtained from CNGBdb with accession number. Genome annotations of *Q. variabilis* are available at FigShare. (10.6084/m9.figshare.23910282). Other data that support the findings of this study are available from the corresponding authors upon reasonable request.

## Author contributions

YW: Conceptualization, Data curation, Formal analysis, Investigation, Validation, Visualization, Writing – original draft, Writing – review & editing. JL: Conceptualization, Data curation, Investigation, Visualization, Writing – review & editing. YC: Data curation, Formal analysis, Investigation, Visualization, Writing – review & editing. ZY: Data curation, Formal analysis, Resources, Visualization, Writing – review & editing. PL: Investigation, Writing – review & editing. GL: Project administration, Resources, Supervision, Writing – review & editing. QY: Conceptualization, Funding acquisition, Methodology, Project administration, Resources, Supervision, Writing – review & editing.
