# Supplementary material for: Genome-wide identification of TCP transcription factors and their potential roles in hydrolyzable tannin production in Quercus variabilis cupule
Source: Front Plant Sci. 2024 Aug 6;15:1444081. doi: 10.3389/fpls.2024.1444081 (PMC11333348; doi:10.3389/fpls.2024.1444081)
Supplement: Supplementary file 1 [file DataSheet_1.docx]

Supplementary Material

**Supplementary Information**

**Table S1** Analysis of physicochemical properties of members of *TCP* gene family in *Quercus variabilis*

| **Gene ID** | **Gene name** | **Number of Amino Acid/aa** | **Molecular Weight/KDa** | **Isoelectric**  **point** | **Instability Index** | **Aliphatic Index** | **Grand Average of Hydropathicity** |
| --- | --- | --- | --- | --- | --- | --- | --- |
| QV_Chr01G01200.1 | QvTCP1 | 470 | 52.55 | 8.28 | 50.39 | 59.13 | -0.700 |
| QV_Chr02G24110.1 | QvTCP2 | 371 | 41.70 | 9.66 | 49.07 | 76.52 | -0.596 |
| QV_Chr02G29390.1 | QvTCP3 | 355 | 39.15 | 8.30 | 49.04 | 59.89 | -0.739 |
| QV_Chr02G31040.1 | QvTCP4 | 319 | 34.49 | 7.32 | 57.55 | 62.76 | -0.813 |
| QV_Chr02G44940.1 | QvTCP5 | 566 | 60.16 | 6.65 | 65.58 | 54.08 | -0.797 |
| QV_Chr03G17410.1 | QvTCP6 | 269 | 28.88 | 7.11 | 50.6 | 63.87 | -0.575 |
| QV_Chr03G23810.1 | QvTCP7 | 408 | 43.26 | 7.88 | 59.45 | 57.43 | -0.554 |
| QV_Chr05G14930.1 | QvTCP8 | 371 | 40.90 | 6.43 | 63.45 | 65.31 | -0.688 |
| QV_Chr05G20540.1 | QvTCP9 | 482 | 52.53 | 7.92 | 53.37 | 58.36 | -0.875 |
| QV_Chr06G24030.1  QV_Chr07G15170.1  QV_Chr07G18690.1  QV_Chr08G10340.1  QV_Chr08G15870.1  QV_Chr08G20600.1  QV_Chr09G08500.1  QV_Chr10G07420.1  QV_Chr10G09400.1  QV_Chr10G10750.1  QV_Chr10G10790.1  QV_Chr10G10800.1  QV_Chr12G06960.1 | QvTCP10  QvTCP11  QvTCP12  QvTCP13  QvTCP14  QvTCP15  QvTCP16  QvTCP17  QvTCP18  QvTCP19  QvTCP20  QvTCP21  QvTCP22 | 264  466  211  430  332  353  380  410  369  287  326  392  381 | 27.59  51.82  22.44  47.23  36.25  37.67  39.77  43.58  40.80  32.74  37.40  44.49  40.37 | 9.72  6.04  8.82  6.39  6.19  8.48  6.26  6.93  8.41  7.21  7.21  6.05  6.46 | 46.32  50.69  58.46  50.26  41.28  64.2  49.35  67.46  49.81  39.71  34.72  50.49  50.56 | 63.33  87.85  68.63  57.47  58.86  71.98  65.76  58.63  65.34  67.28  71.5  56.71  63.23 | -0.513  -0.226  -0.364  -0.741  -0.724  -0.305  -0.515  -0.676  -0.613  -0.847  -0.745  -0.930  -0.497 |

**Table S2** Oligonucleotide primer sequences used for qRT-PCR and in Chinese cork oak experiments

| **Name** | **Forward primer** | **Reverse primer** |
| --- | --- | --- |
| *QvTCP3 SK* | 5´GCGGCCGCTCTAGAACTAGTGATGATATCAAGGTCAAAGGAAGCA 3´ | 5´GGTCGACGGTATCGATAAGCTTCATTTTGAAGGAAACTCTTGGTC 3´ |
| *QvTCP6 SK* | 5´GCGGCCGCTCTAGAACTAGTGATGGCAGAAAACAAGCCTGCAG 3´ | 5´GGTCGACGGTATCGATAAGCTCTACTGCCTTGATTCTTCAGAG 3´ |

**Table S3** Table of information on covariance blocks of *Quercus variabilis* TCP family members

| **Region -1** | | | **Region -2** | | |
| --- | --- | --- | --- | --- | --- |
| **Chromosome** | **Initiation site** | **Termination site** | **Chromosome** | **Initiation site** | **Termination site** |
| Chr1 | 2600103 | 2601515 | Chr10 | 22655867 | 22657107 |
| Chr10 | 19179831 | 19182299 | Chr2 | 65770354 | 65772479 |
| Chr10 | 15546075 | 15547881 | Chr3 | 65735835 | 65737530 |
| Chr12 | 14246632 | 14248355 | Chr8 | 48867003 | 48869067 |
| Chr12 | 14246632 | 14248355 | Chr9 | 17008211 | 17009928 |
| Chr2 | 68803838 | 68805951 | Chr3 | 50813524 | 50817894 |
| Chr5 | 46956694 | 46958716 | Chr8 | 26339532 | 26342686 |
| Chr8 | 26339532 | 26342686 | Chr8 | 38827850 | 38828848 |

**Table S4** Table of information on covariate gene pairs of *Quercus variabilis* TCP family members

| **Gene** | **Ka** | **Ks** | **Ka/Ks** |
| --- | --- | --- | --- |
| *QVTCP1 - QVTCP19* | 0.5535 | 3.1367 | 0.1765 |
| *QVTCP18 -* *QVTCP3* | 0.4130 | 1.1794 | 0.3502 |
| *QVTCP17 - QVTCP7* | 0.2606 | 1.3591 | 0.1918 |
| *QVTCP22 - QVTCP15* | 0.3991 | 1.5226 | 0.2621 |
| *QVTCP22 - QVTCP16* | 0.5049 | 2.2029 | 0.2292 |
| *QVTCP4 - QVTCP6* | 0.2203 | 1.8845 | 0.1169 |
| *QVTCP8 - QVTCP13* | 0.4451 | 2.2397 | 0.1987 |
| *QVTCP13 - QVTCP14* | 0.3044 | 2.4938 | 0.1221 |


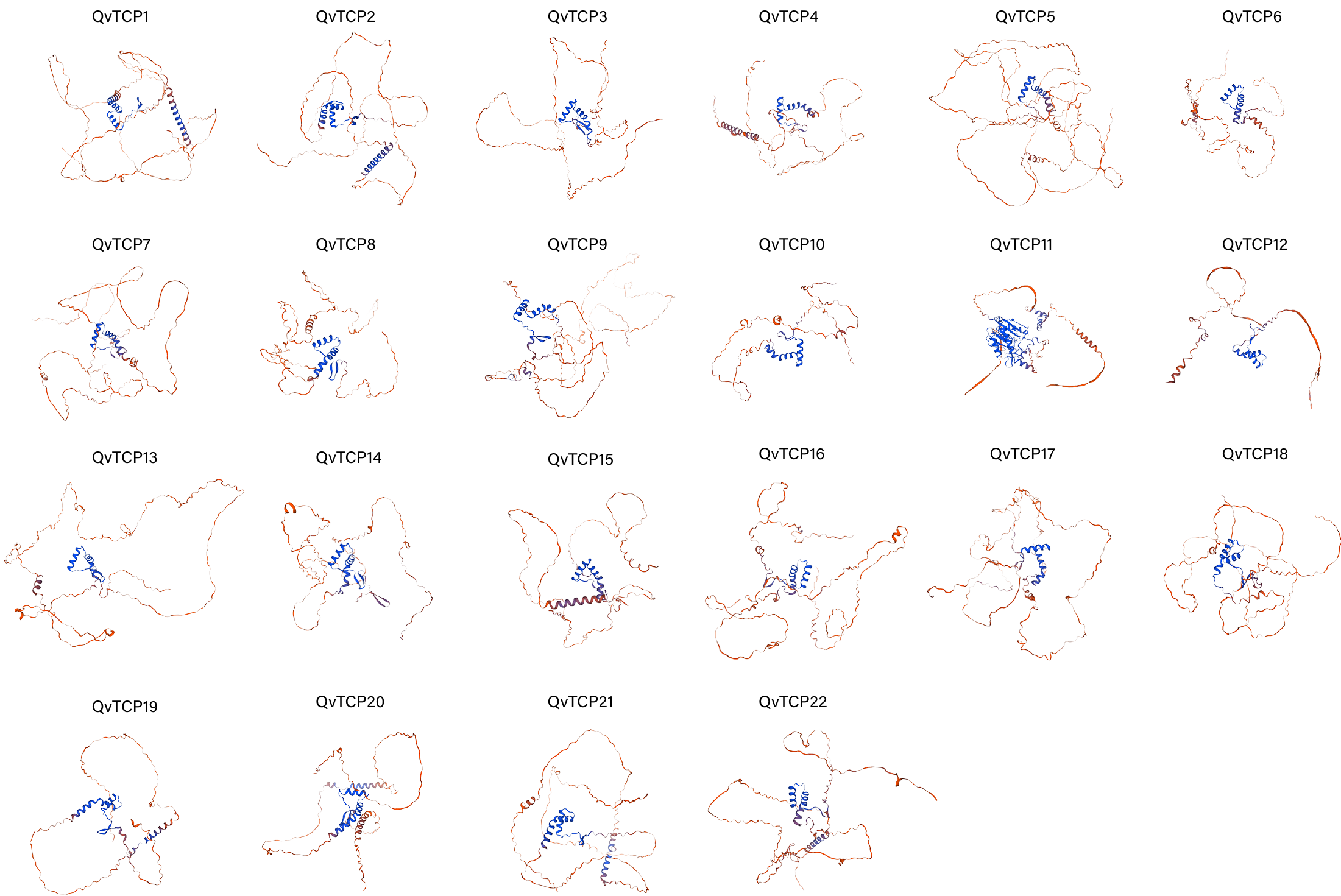


Figure S1 Predicted protein structure models of TCP Family in *Q. variabilis*. The predicted protein structure models of the TCP family in *Q. variabilis* are displayed. The blue color signifies regions where the prediction results are considered highly reliable, while the red color signifies regions with lower reliability in the prediction results.
